# Nerve Transplantation from Animal to Man

**Published:** 1888-07

**Authors:** 


### Nerve Transplantation from Animal to Man.

A novel operation is reported from Vienna as having been performed by Dr. Gersung on the person of Prof. Von Fleischl, who accidentally wounded himself sixteen years ago while performing a post-mortem examination. The result was severe inflammation of the whole right upper limb, which was followed by the formation of neuromata on various branches of the median nerve, to relieve the pain of which, resection of the nerve at various sites was resorted to, causing anæsthesia of the parts supplied by it. Fresh neuromata, however, occurred, and finally the pain became so severe that a further operation became necessary. On March 4th the patient was anæsthetized, and the neuroma, which was situated behind the volar-carpal ligament, was excised. A piece of the sciatic nerve of a freshly-killed rabbit was then obtained and sutured to the ends of the cut nerve, and to the connective tissue between. Healing took place by first intention, and as two months have now elapsed since the date of operation, and the pain has not returned, it is hoped that the so-far favorable result will prove to be permanent. Sensation is gradually returning in the anæsthetic areas. The success of this novel attempt shows that surgeons have not yet attained the limits of the possible in this department.—*Med. Press and Circular.*

